# Supplementary material for: The interactome of intact mitochondria by cross-linking mass spectrometry provides evidence for coexisting respiratory supercomplexes
Source: Mol Cell Proteomics. 2017 Dec 8;17(2):216–32. doi: 10.1074/mcp.RA117.000470 (PMC5795388; doi:10.1074/mcp.RA117.000470)
Supplement: Supplemental Data [file supp_RA117.000470_133922_0_supp_23978_fzffwf.zip › spectra_annotation/mito_spectra_annotation/74-1-3-1-22-1.pdf]

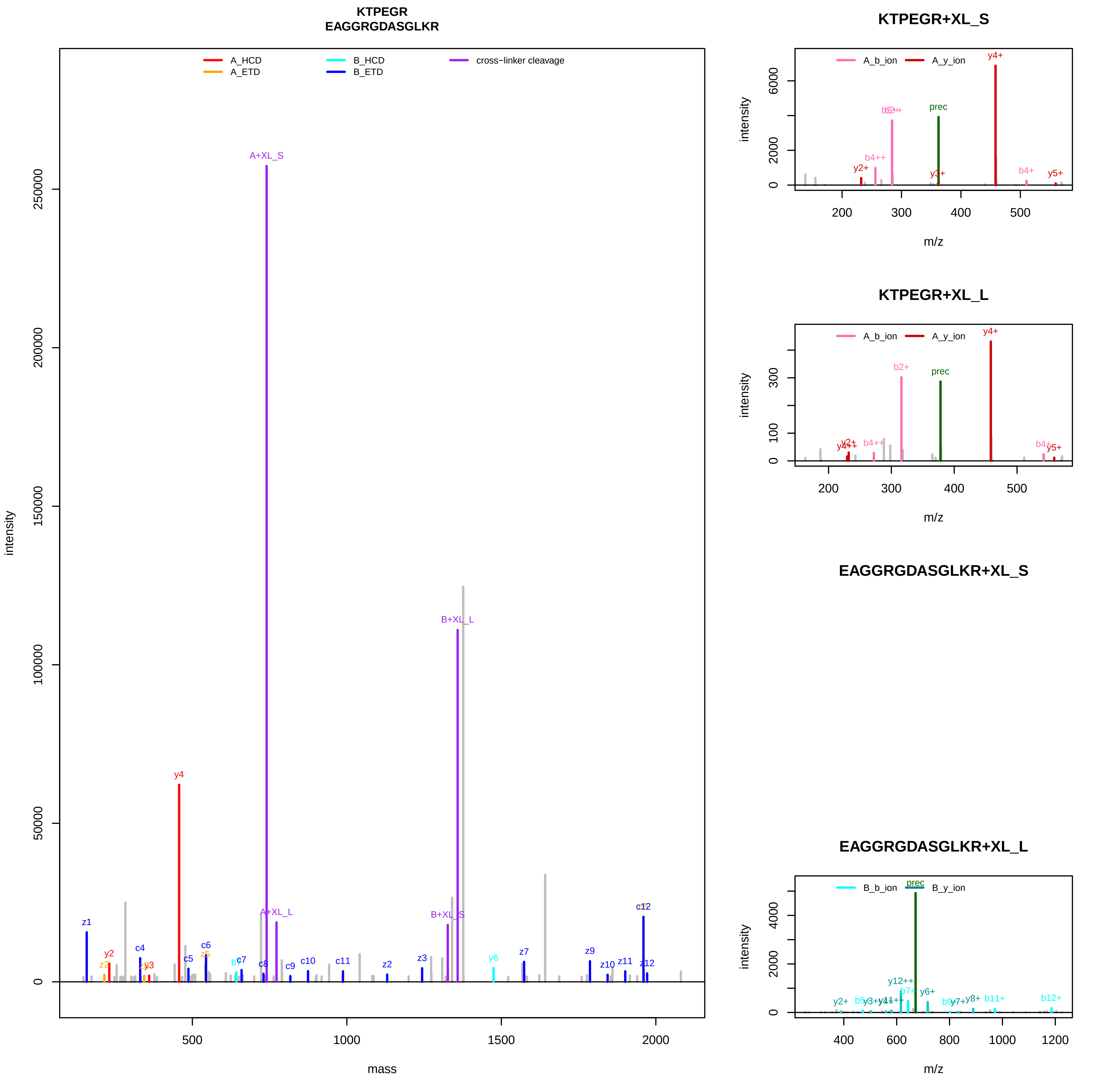
KTPEGR
EAGGRGDASGLKR
A_HCD
A_ETD
B_HCD
B_ETD
cross−linker cleavage
A+XL_S
B+XL_L
A+XL_L
B+XL_S
y4
y2
z1
c4
c5
c6
c7
c8
c9
c10
c11
z2
z3
y6
z7
z9
z10
z11
c12
z12
intensity
mass
0
50000
100000
150000
200000
250000
500
1000
1500
2000
KTPEGR+XL_S
A_b_ion
A_y_ion
y4+
prec
b4++
y2+
y3+
b4+
y5+
m/z
KTPEGR+XL_L
b2+
EAGGRGDASGLKR+XL_S
EAGGRGDASGLKR+XL_L
B_b_ion
B_y_ion
y12++
y6+
y8+
b11+
b12+
y2+
